# C-Reactive Protein Promotes the Expansion of Myeloid Derived Cells With Suppressor Functions

**DOI:** 10.3389/fimmu.2019.02183

**Published:** 2019-09-18

**Authors:** Rachel V. Jimenez, Valeriya Kuznetsova, Ashley N. Connelly, Zdenek Hel, Alexander J. Szalai

**Affiliations:** ^1^Division of Clinical Immunology and Rheumatology, Department of Medicine, University of Alabama at Birmingham, Birmingham, AL, United States; ^2^Department of Pathology, University of Alabama at Birmingham, Birmingham, AL, United States; ^3^Department of Microbiology, University of Alabama at Birmingham, Birmingham, AL, United States

**Keywords:** acute phase response, inflammation, myelopoiesis, innate immunity, cancer

## Abstract

Previously we established that human C-reactive protein (CRP) exacerbates mouse acute kidney injury and that the effect was associated with heightened renal accumulation of myeloid derived cells with suppressor functions (MDSC). Herein we provide direct evidence that CRP modulates the development and suppressive actions of MDSCs *in vitro*. We demonstrate that CRP dose-dependently increases the generation of MDSC from wild type mouse bone marrow progenitors and enhances MDSC production of intracellular reactive oxygen species (iROS). When added to co-cultures, CRP significantly enhanced the ability of MDSCs to suppress CD3/CD28-stimulated T cell proliferation. Experiments using MDSCs from FcγRIIB deficient mice (FcγRIIB^−/−^) showed that CRP's ability to expand MDSCs and trigger their increased production of iROS was FcγRIIB-independent, whereas its ability to enhance the MDSC T cell suppressive action was FcγRIIB-dependent. Importantly, CRP also enabled freshly isolated primary human neutrophils to suppress proliferation of autologous T cells. These findings suggest that CRP might be an endogenous regulator of MDSC numbers and actions *in vivo*.

## Introduction

Human C-reactive protein (CRP) is the prototypical acute phase reactant; CRP serum levels can rapidly increase from typically ≤3 μg/ml at baseline to upwards of 500 μg/ml in response to pro-inflammatory cytokines produced during inflammation (e.g., IL-6, IL-1β, and TNFα) (1, 2). The human CRP molecule is a planar, pentameric pattern-recognition receptor with a high affinity for phosphocholine (3) that can function as an opsonin (4, 5), activate the classical pathway of complement (6), and bind to various Fc receptors (FcR) thereby triggering effector responses like phagocytosis and cytokine secretion (7, 8). Its wide-ranging blood levels and sensitivity to inflammation make human CRP a useful clinical biomarker of diseases such as cardiovascular, autoimmune, and Alzheimer's disease (9, 10). For example, CRP levels are often monitored in patients with acute kidney injury (AKI) wherein they correlate with increased AKI risk, severity, and clinical outcomes (11–13). Importantly, our group recently established that expression of human CRP (by CRP transgenic mice; CRPtg) exacerbated renal ischemia reperfusion injury, an experimental model of AKI (14). Notably, the detrimental action of CRP was associated with an increased renal accumulation of myeloid cells with a suppressor phenotype (hereafter, MDSC). Moreover, we showed that antibody-mediated depletion of MDSCs alleviated renal injury in CRPtg and that targeted lowering of human CRP, which led to diminished MDSC renal accumulation, lessened the severity of AKI in CRPtg (15).

Neither mouse nor human MDSCs have a unique marker and their exact origins remain equivocal; however, there is a growing consensus that MDSCs are a heterogeneous group of immature and highly proliferative cells that arise in various pathological states (16, 17). As their name implies, MDSCs potently suppress the proliferation of cells in their immediate vicinity; suppression of T cell proliferation being the gold standard by which this is assessed. The suppressive action of MDSCs is thought to be the consequence of their ability to deplete the essential amino acids arginine (achieved *via* MDSC expression of arginase) and tryptophan (achieved *via* MDSC expression of indolamine-2,3-dioxygenase), and by their robust production of reactive nitrogen and oxygen species (RNS and ROS, respectively) (18). Although MDSCs were initially described as key mediators of immune suppression during tumorigenesis (19), it is increasingly evident that MDSCs also participate during trauma (20, 21) and sepsis (22, 23).

To understand how human CRP might impact the biology of MDSCs, and thereby better understand the sequence of events that leads to worsening of AKI in CRPtg mice, herein we used mouse bone marrow cultures to directly interrogate the impact of human CRP on mouse MDSC development and suppressive actions. Since any observed effect of human CRP on mouse MDSCs might be an aberration of the xenogeneic (i.e., human protein/mouse cell) system, we also performed studies using freshly isolated primary human myeloid cells. Our results show that in the presence of human CRP, mouse bone marrow myeloid progenitor cell commitment is biased toward MDSCs and away from dendritic cells (DC). Furthermore, human CRP triggers the generation of iROS by mouse MDSCs and enhances their ability to suppress the proliferation of CD3/CD28 stimulated mouse CD4^+^ T cells. Interestingly, human CRP triggered enhancement of the immune suppressive action of mouse MDSCs is FcγRIIB-dependent, but its ability to stimulate iROS is not. Human CRP also augmented the production of iROS by freshly isolated human peripheral blood neutrophils and enabled them to exert a suppressive effect on the proliferation of autologous human T cells. Our findings demonstrate that CRP might be an endogenous regulator of MDSCs and suggest that monitoring and/or targeting CRP might be a useful clinical strategy for a growing list of pathologies in which MDSCs are known to participate.

## Materials and Methods

### Mice

All animal use protocols were approved by the Institutional Animal Care and Use Committee at the University of Alabama at Birmingham and were consistent with the *Guide for the Care and Use of Laboratory Animals; Eighth Edition* (NIH Academies Press, 2011). All mice used were from the C57BL/6 background, housed in the same vivarium at constant humidity (60 ± 5%) and temperature (24 ± 1°C) with a 12 h light cycle (6 a.m.−6 p.m.), and maintained *ad libitum* on sterile water and regular chow (Harlan Teklad). Mice were at least 8 weeks old when bone marrow, spleens, and lymph nodes were harvested and both sexes were used. Where indicated, bone marrow (BM) was harvested from FcγRIIB deficient mice (FcγRIIB^−/−^; B6.129S4-Fcgr2b^tm1TtK^ N12, Taconic Farms model 580) that lack functional expression of the gene encoding the α-chain of mouse FcγRIIB (24).

### Generation of Mouse Myeloid Derived Suppressor Cells

Mouse bone marrow myeloid derived suppressor cells (BM-MDSC) were generated as described by Höchst et al. (25). Briefly, BM was flushed from mouse femurs and tibias using a Hank's Balanced Salt Solution (HBSS; Gibco) filled 1 ml syringe fitted with a 25G × 5/8^″^ needle. The recovered bone marrow was strained through a nylon filter (70 μm) and erythrocytes were lysed (Hybri-Max Red Blood Cell Lysing Buffer; Sigma R7757). Filtered BM cells were suspended in Minimum Essential Medium Eagle—Alpha Modification (αMEM; Lonza 12-169F) supplemented with 10% heat inactivated-fetal bovine serum (HI-FBS, Gibco 10082147), 2 mM GlutaMAX (Gibco 35050061), 100 U per ml/100 μg per ml penicillin/streptomycin (Gibco 15140122), 1 mM sodium pyruvate (Gibco 11360070), 55 μM β-mercaptoethanol (Gibco 21985023), and 40 ng/ml mouse granulocyte/macrophage-colony stimulating factor (GMCSF; Shenandoah Biotechnology 200-15) and then seeded into 12-well tissue culture-treated plates (1 × 10^6^ cells/well) and grown (37°C, 5% CO_2_) for 96 h (i.e., d4) unless otherwise noted. The culture medium was changed at 72 h (d3). Highly purified (~95%) human CRP from pleural/ascites fluids (US Biological Sciences C7907-26A) was filtered (0.2 μm) and diluted in Tris-buffered saline pH 7.4 without preservatives, and added at the start of culture (d0) and with the media change at 72 h. Prior to use, samples of human CRP were subjected to polyacrylamide gel electrophoresis to confirm its integrity (data not shown). On d4, cells were harvested with a cell scraper for cytometry analysis or used in downstream experiments as otherwise described. To negatively enrich MDSCs, BM-MDSCs (d4 cultures) were subjected to separation using the EasySep mouse CD11c positive selection kit II (Stemcell Technologies 18780) according to the manufacturer's instructions. This approach effectively removed the contaminating CD11c^+^ fraction (i.e., DCs), yielding a highly purified (94 ± 1.5%) CD11c^−^ MDSC fraction (see Supplemental Figure 1). For experiments utilizing FcγRIIB^−/−^ BM-MDSCs, wild type BM-MDSCs were grown concomitantly and used simultaneously in suppression assays or ROS assays.

### Cell Cycling Analysis by Bromodeoxyuridine Incorporation

To assess cell cycling d4 BM-MDSC were exposed to human CRP for 24 h, with 20 μM bromodeoxyuridine (BrdU; Sigma B5002) added 3 h prior to harvesting cells with a cell scraper. BM-MDSCs were then fixed using pre-chilled 70% ethanol added dropwise while vortexing. After incubation for 20 min. the DNA was linearized by adding 2N HCl while vortexing. After incubation for 20 min. the cells were permeabilized with 0.1 M Na_2_B_4_O_7_ for 2 min. Next, non-specific binding was blocked by 15 min incubation (4°C) with anti-CD16/CD32 monoclonal antibody (mAb clone 93; eBioscience 14-0161-82), and finally BrdU incorporation was probed using of APC conjugated anti-BrdU antibody (clone Bu20a, BioLegend 339808) for 30 min at 4°C (4 μl/tube). Ten minutes prior to cell cytometry total DNA was stained with 1 μg of 7-aminoactinomycin D (7-AAD, Invitrogen A1310). Cell cytometry was performed on a BD LSRII cytometer using BD FACSDiva version 6.1.3 software, a standard gating strategy was used to identify cells in the G0/G1, S, and G2/M phases of cell division [adapted from (26)], and the acquired data was analyzed using FlowJo version 10.3. Briefly, single cells were gated on using an SSC-A × SSC-H dot plot and apoptotic cells (7-AAD^lo^) were excluded. Untreated live cells were used to gate on cells in the G0/G1 (7-AAD^int^BrdU^lo^), S (7-AAD^lo−hi^BrdU^+^), and G2/M (7-AAD^hi^BrdU^+^) phases of cell division. Cells in S phase were further subdivided into three subpopulations corresponding to cells in early (7-AAD^lo^BrdU^+^), middle (7-AAD^int^BrdU^+^), and late S phase (7-AAD^hi^BrdU^+^).

### Mouse MDSC-Mediated Mouse T Cell Suppression Assays

To isolate mouse CD4^+^ T cells the spleen and lymph nodes (inguinal, axillary, brachial) from wild type mice were mechanically homogenized, erythrocytes were lysed, and the resultant homogenate filtered (70 μm). The single cell suspension was then subjected to negative selection using the EasySep mouse CD4^+^ T cell isolation kit (Stemcell Technologies 19852) according to the manufacturer's instructions. Negatively enriched CD4^+^ T cells were then stained with 0.5 μM carboxyfluorescein succinimidyl ester (CFSE; Invitrogen 65085084) in PBS for 20 min at room temperature, washed and re-suspended in RPMI 1640 media (Gibco 11875119) supplemented with 5% HI-FBS, 2 mM GlutaMAX, 100 U/ml penicillin, 100 μg/ml/streptomycin, 1X MEM non-essential amino acids (Gibco 219850232) and 55 μM β-mercaptoethanol. Mouse CFSE^+^CD4^+^ T cells were then added to a tissue culture-treated 96-well plate (2 × 10^5^ cells/well) coated with 2 μg/ml of anti-CD3ϵ mAb (functional grade, clone 145-2C11; Invitrogen 16-0031-82) in the presence of 1 μg/ml soluble anti-CD28 mAb (functional grade, clone 37.51; Invitrogen 16-0281-81). After 72 h (d3), mouse CD4^+^ T cells were harvested and their proliferation (CFSE dilution) was assessed by flow cytometry (Supplemental Figures 2A,B). Prior to performing T cell suppression assays each lot of anti-CD3ε mAb was titrated and used at concentrations that resulted in 3–5 discernable generations of CFSE^+^CD4^+^ T cells after 72 h of culture (Supplemental Figure 2B). When studying the effects of MDSCs on T cell proliferation MDSCs were from d4 cultures and they were added to the T cells to achieve effector:target (E:T) ratios ranging from 10E:1T to 1E:20T. When studying the effects of CRP on MDSC-mediated suppression of T cell proliferation CRP (1–100 μg/ml) was added only at the beginning of co-culture. Proliferation of mouse CFSE^+^CD4^+^ T cells was assessed and is reported following standard conventions detailed by Roederer (27). Thus, (i) when non-proliferated and proliferating generations were discrete and easily discerned (e.g., Figure 3D) we calculated the proliferation index, i.e., a ratio of the average number (across biological and technical replicates) of generations of proliferating T cells normalized to the maximum number of generations of proliferating T cells when they were cultured in isolation, (ii) when non-proliferated cells were in excess (e.g., Figure 3D) we calculated the division index, i.e., the average number of cell divisions carried out by all the T cells in co-cultures, normalized to their maximum number of cell divisions when they were cultured in isolation, and (iii) in cases where proliferating generations of T cells were difficult to resolve (due to high intergeneration variance or high autofluorescence; e.g., Figure 5C) we calculated the fraction diluted, i.e., we averaged the fraction of T cells in the final culture that divided at least once and normalized this to maximum proliferation achieved by T cells cultured in isolation or to co-cultures without human CRP added, depending on the experiment. Proliferation and division indexes and the fraction diluted were calculated using CFSE dilution histograms normalized to mode (the most populous T cell generation) as defined by FlowJo version 10.3. In all cases, the experiments were conducted with technical triplicates to ensure the rigor of the co-culture system.

### Human Neutrophil-Mediated Human T Cell Suppression Assays

Under the auspices of protocols approved by the Institutional Review Board of the University of Alabama at Birmingham, in accordance with the recommendations of the Belmont Report, and after subjects gave written informed consent, neutrophils were purified from the whole blood of healthy adult human donors using the EasySep Direct Human Neutrophil Isolation Kit (StemCell Technologies 19666) according to the manufacturer's instructions. Concurrently, autologous human CD3^+^ T cells were isolated from PBMCs by immunomagnetic negative selection using EasySep Human T Cell Isolation Kit (Stemcell Technologies 17951) according to the manufacturer's protocol. The isolated CD3^+^ T cells were stained with 1.25 μM CFSE (Invitrogen C34554) in PBS for 8 min at 37°C. To stimulate their proliferation, 5 × 10^4^ CFSE^+^CD3^+^ human T cells in RPMI1640 (Corning 10-040-CM) supplemented with 10% heat inactivated human serum type AB (Atlanta Biologicals S40110) and 1X penicillin/streptomycin (Corning 30-002-CI) were added to a 96-well plate coated with 5 μg/ml anti-CD3ε mAb (BioLegend 300401) and 2 μg/ml soluble anti-CD28 mAb (BioLegend 302901). Autologous neutrophils were added to the T cells to achieve a 1:1 E:T ratio and the cells were incubated at 37°C in 5% CO_2_ for 72, 96, or 120 h. Human CD3^+^ T cell proliferation (CFSE dilution) was recorded on an Invitrogen Attune NxT flow cytometer and quantitated as described for mouse T cells.

### Flow Cytometry of Mouse Cells

For mouse BM-MDSC phenotyping and mouse T cell suppression assays single cell suspensions were stained with eFluor780 viability dye (eBioscience 65-0865) for 30 min at room temperature, fixed with 0.5X Fixation Buffer (BioLegend 420801) for 10 min at room temperature, and non-specific binding was blocked with an anti-CD16/CD32 mAb (clone 93; eBioscience 14-0161-82) for 15 min at 4°C. Cells were then stained for 30 min at 4°C with specific fluorochrome-labeled antibodies (all from BioLegend). Mouse BM-MDSCs were stained using anti-mouse CD11b (clone M1/70), CD11c (clone N418), F4/80 (clone BM8), Ly6C (clone HK1.4), and Ly6G (clone 1A8). Mouse T cells were stained with anti-mouse CD4 (clone RM4-5). After staining the cells were washed and suspended in PBS and cytometry performed on a BD LSR-II cytometer equipped with BD FACSDiva version 6.1.3. The acquired data were analyzed with FlowJo version 10.3. Debris, doublets, and eFlour780^+^ dead cells were gated out before any assay-specific gating (gating strategy shown in Supplemental Figure 2A).

### Assessment of Reactive Oxygen Species Production

To measure extracellular ROS, mouse BM-MDSCs (d4) were harvested or primary human neutrophils were isolated and added (5 × 10^5^/well) to a white 96-well plate (Corning 3355) containing 200 μM luminol (Sigma A8511) and 1.6 U/ml of horseradish peroxidase (Sigma P2088). The cells were thereafter left untreated (HBSS control) or treated with human CRP or 100 nM phorbol 12-myristate 13-acetate (PMA) and the amount of oxidized luminol (luminescence units, LU) measured immediately and for up to 60 min thereafter on a Bio-Tek Synergy 2 with Gen5 version 1.10. For each condition the background signal (the first LU reading) was subtracted from all subsequent readings, and for PMA- and CRP-treated cells the data were normalized to their genotype-matched PBS controls (relative LU, RLU). To measure iROS, 5 × 10^5^ enriched mouse MDSCs (WT or FcγRIIB^−/−^) or primary human neutrophils were added to the wells of a tissue culture-treated, clear-bottom, black-sided 96-well plate (Greiner Bio-One 65509099) loaded with freshly reconstituted 2.5 μM 2′,7′-dichlorodihydrofluorescein diacetate (H_2_DCFDA, Invitrogen D399) at 37°C in 5% CO_2_ for 30 min. H_2_DCFDA is cell-permeant and cleaved intracellularly, preventing its exit, and specifically fluoresces upon oxidation by iROS. Thereafter the cells were left untreated (PBS control) or treated with human CRP or 100 nM PMA and fluorescence intensity (FI; excitation 485 nm/emission 535 nm) was immediately measured and for 60–180 min thereafter on a Tecan Infinite M200 Pro using i-control version 1.7.1.12. For each condition the background signal (the first FI reading) was subtracted from all subsequent readings, and for PMA- and CRP-treated cells the data were normalized to their genotype-matched HBSS/PBS controls (relative FI, RFI).

### Statistical Analysis

Raw data from biological replicates (experiments using BM cultures from different mice) and technical replicates (experiments repeated using a single BM cultures) were pooled as appropriate and the means with associated SEMs or SDs is presented. Group comparisons were done using one-way or two-way analysis of variance (*ANOVA*) followed by *post-hoc* Dunnett's or Sidak's multiple comparisons tests, or using 1-tailed Student's *t*-tests (un-paired or paired as appropriate). Differences were considered significant when the test *p* ≤ 0.05. To estimate CRP's potency we employed non-linear regression to estimate the concentration of CRP required to enhance MDSC mediated suppression of T cell proliferation by 50% (*IC*_50_). All statistical and regression analyses were done using GraphPad Prism version 7.00.

## Results

### Human CRP Promotes the Generation of Mouse MDSCs

**Figure 1:**
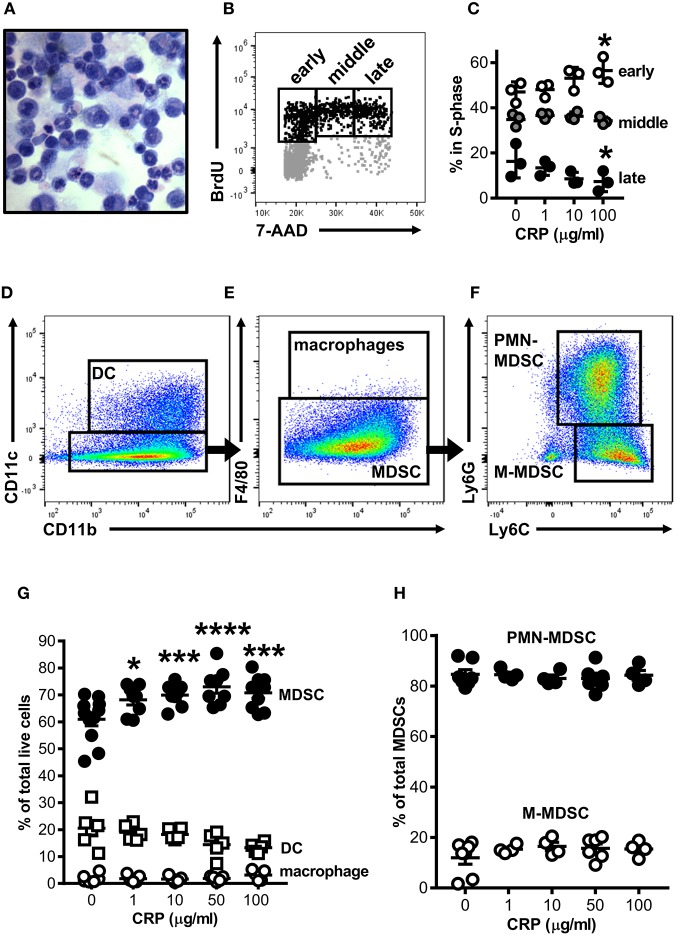
Human CRP promotes expansion of mouse MDSCs. Mouse bone marrow progenitors cultured under conditions tailored to generate MDSCs (25) were used to study the effects of human CRP on MDSC expansion. After 4 days in culture (d4) the majority of cells retained their immature nuclear morphologies (**A**; light micrograph showing Wright-Giemsa and hematoxylin stained cytospin; 100X) and 25 ± 2.1% were in the S-phase of cell division (**B**; representative flow cytometry dot plot showing the gating strategy used to identify cells in the early, middle, and late stages of S-phase; see the *Materials and Methods* for details). Addition of human CRP to the cultures dose-dependently increased the entry of cells into early S-phase **(C)**; the asterisks indicate *p* < 0.05 for Dunnett's tests comparing cultures receiving 100 μg/ml CRP and those left untreated. The representative data shown is the mean ± SD for technical triplicates (1 of 3 biological replicates). **(D–F)** Flow cytometry gating strategy used for identification of CD11c^+^CD11b^+^ DCs **(D)**, CD11b^+^CD11c^−^F4/80^+^ macrophages **(E)**, and CD11c^−^CD11b^+^F4/80^−^Ly6G^+^Ly6C^+^ MDSCs **(E)**; MDSCs were further divided into PMN-MDSC (CD11b^+^CD11c^−^F4/80^−^Ly6G^+^Ly6C^+^) or M-MDSC (CD11b^+^CD11c^−^F4/80^−^Ly6G^−^Ly6C^+^) **(F)**. Human CRP dose-dependently promotes the expansion of MDSCs **(G)** but does not affect the relative proportion of PMN- and M-MDSC subtypes **(H)**. In **(G,H)**, the mean ± SEM for 6–11 separate cultures are shown. The asterisks indicate ^*^p < 0.01, ^***^p < 0.005, and ^****^p < 0.0001 for Dunnett's tests comparing cultures that received CRP to those that did not.

Mouse bone marrow (BM) was grown under conditions previously shown to expand myeloid derived cells into cells with suppressor functions [i.e., BM-MDSCs; protocol adapted from Hochst et al. (25)]. After 4 days in culture the majority of cells recovered either retained an immature myelocyte mononuclear appearance or had ring-shaped nuclei, while fewer had a polymorphonuclear appearance typical of mature granulocytes (Figure 1A); this heterogeneity is consistent with the reported range of nuclear morphologies characteristic of MDSCs found *in vivo* in both mice and humans (28). To determine the impact of CRP on the growth of mouse bone marrow progenitors, human CRP was added to cultures and their cell cycling was assessed by flow cytometry after BrdU/7-AAD incorporation. We found that the addition of human CRP increased the frequency of cells entering early S-phase (Figure 1B); this effect was dose-dependent and achieved statistical significance at a dose of 100 μg/ml of CRP (Figure 1C). Additional experiments showed that the CRP dependent increase in the number of cells entering S-phase was not an artifact due to selective culling of cells exposed to CRP, as CRP treatment had no statistically significant effect on (i) the total number of cells recovered on day 4 of culture, (ii) the proportion of apoptotic and necrotic cells on day 4 of culture, and (iii) the overall viability of cells recovered on day 4 of culture (assessed by hemocytometer counts of trypan blue negative cells, flow cytometry frequencies of Annexin V^+/−^7-AAD^+^ cells, and flow cytometry frequencies of viability dye eFlour780^low^ cells, respectively) (data not shown).

By flow cytometry the majority of cells in mouse BM-MDSC cultures (66.3 ± 3.2% of cells recovered from *n* = 12 cultures) displayed a CD11b^+^CD11c^−^F4/80^−^Ly6G^+^Ly6C^+^ MDSC surface phenotype (Figures 1D,E); 84.6 ± 5.0% of these were of the Ly6G^+^Ly6C^+^ polymorphonuclear MDSC subtype (PMN-MDSC) and 12.0 ± 6.7% were the Ly6G^−^Ly6C^+^ monocytic MDSC subtype (M-MDSC) (Figure 1F). The remaining cells in d4 cultures were CD11b^+^CD11c^+^ DCs (24.0 ± 10.5% of all cells in culture) or CD11b^+^CD11c^−^F4/80^+^ macrophages (1.7 ± 1.5% of all cells in culture) (Figures 1D–F). As there is no accepted marker for MDSCs, we determined that d4 cultures contained a preponderance of MDSCs as verified by direct hemocytometer counts of fractions captured vs. not captured by CD11c positive immunomagnetic selection (fractionations of *n* = 7 separate cultures). By this approach it was estimated that 76.7 ± 7.1% of all cells in the d4 cultures were CD11c^−^ MDSCs (Supplemental Figures 1D–F), slightly more than estimated by flow cytometry (66.3 ± 3.2%). Importantly, after immunomagnetic removal of contaminating CD11c^+^ cells the remaining cells were ~94% pure MDSCs as determined by flow cytometry (Supplemental Figures 1E,F).

When added to the BM cultures, human CRP significantly and dose-dependently increased the proportion of MDSCs generated (Figure 1G). Notably the observed increase in MDSCs was at the expense of DCs, whose numbers were decreased by addition of CRP [as we have described elsewhere (29)]. Accordingly, the observed CRP dependent increase in the number of BM cells entering S-phase (Figures 1B,C) can be explained by CRP's selective enhancement of MDSC proliferation. However, although human CRP selectively promoted MDSC generation (Figure 1G), CRP had no effect on the relative proportion of PMN- vs. M-MDSCs (~85 and ~15%, respectively; Figures 1F,H). These data show that under conditions known to expand MDSCs from BM precursors, CRP selectively potentiates the expansion of cells with an MDSC surface phenotype.

### Human CRP Augments Mouse MDSC Production of Intracellular Reactive Oxygen Species

**Figure 2:**
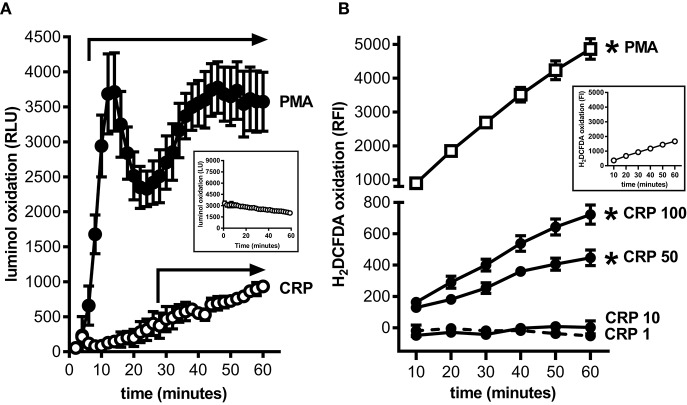
Human CRP triggers intracellular reactive oxygen species generation by mouse MDSCs. Mouse bone marrow progenitors cultured under conditions tailored to generate MDSCs (25) were used to study the effects of human CRP on ROS generation. **(A)** ROS generated by BM-MDSCs stimulated with PMA (100 nM) vs. human CRP (100 μg/ml) was measured by luminol assay. PMA stimulated BM-MDSCs exhibited an initial respiratory burst followed by sustained ROS production. In contrast BM-MDSCs stimulated with CRP showed a monophasic increase in ROS with no early respiratory burst. The data shown are the mean ± SEM for three biological replicates each done with technical triplicates. The horizontal arrows begin at the first time-point when luminol oxidation (relative luminescence units, RLU, as defined in the *Materials and Methods*) was significantly elevated compared to the untreated cells (*p* < 0.05 for Sidak's multiple comparisons tests; LU for untreated cells shown in the inset). **(B)** Intracellular ROS generated by mouse MDSCs (enriched by depletion of CD11c^+^ cells; see the *Materials and Methods* and Supplemental Figure 1) stimulated with PMA (100 nM) vs. human CRP (1–100 μg/ml) was measured with the cell-permeant redox-sensing fluorescent dye H_2_DCFDA. Mouse MDSCs exhibited a significant increase in iROS-dependent fluorescence intensity when stimulated with 100 nM PMA or high concentrations of human CRP. MDSCs stimulated with high amounts of CRP showed substantial generation of iROS. The data shown are the mean ± SEM for three biological replicates each done with five technical replicates, and the asterisks indicate *p* < 0.05 for Sidak's multiple comparisons tests comparing relative fluorescence intensity (RFI, as defined in the *Materials and Methods*) to the untreated cells shown in the inset.

MDSCs are prolific producers of ROS and this supports their capacity to strongly suppress the proliferation of T cells (18). Using a luminol based assay we confirmed that mouse BM-MDSCs generated ROS robustly and in a biphasic pattern when stimulated with PMA (Figure 2A); this likely reflects an initial respiratory burst followed by sustained ROS production. In contrast, mouse BM-MDSCs stimulated with human CRP showed only a monophasic increase in ROS without evidence of a respiratory burst after (Figure 2A), and human CRP did not augment the respiratory burst triggered by PMA (data not shown). We also used the cell-permeant dye H_2_DCFDA to specifically measure the production of intracellular ROS (iROS). Using this approach, we found that human CRP (50 and 100 μg/ml) significantly increased iROS production by enriched mouse MDSCs (Figure 2B). These data show that the mouse BM-MDSCs we generated are capable of robustly producing ROS, and that human CRP at concentrations seen during inflammation (2) specifically increases their iROS.

### Human CRP Augments Mouse MDSC Mediated Immune Suppression

**Figure 3:**
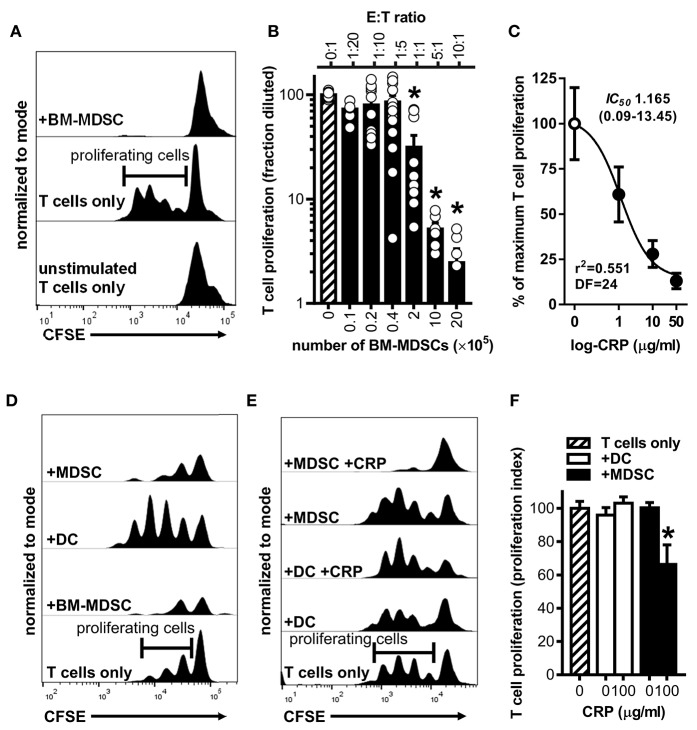
Human CRP enhances the suppressive capacity of mouse MDSCs. Mouse MDSCs were used in co-cultures with mouse T cells to assess their suppressive capacity and the effect of human CRP on MDSC mediated immune suppression. **(A)** Representative CFSE dilution histograms (normalized to mode as defined in the *Materials and Methods*) for CD4^+^ T cells cultured without stimulation and in isolation (bottom trace), cultured with stimulating anti-CD3 and anti-CD28 mAbs but in isolation (middle trace; horizontal gate used to demarcate proliferating cells is shown), or co-cultured with BM-MDSCs (10E:1T) in the presence of stimulating anti-CD3 and anti-CD28 mAbs (top trace). Note that in the presence of BM-MDSCs proliferation of T cells is suppressed. See Supplemental Figure 2 for additional details. **(B)** Pooled results (mean + SEM fraction diluted for *n* = 2–5 co-cultures each conducted in triplicate) from experiments performed with (i) co-cultures of BM-MDSCs plus CFSE-labeled T cells in the presence of stimulating anti-CD3 and anti-CD28 mAbs (solid bars; experiments done as in the top trace of **(A)** but with increasing E:T ratios) or with (ii) CFSE-labeled T cells cultured without BM-MDSCs but with stimulating anti-CD3 and anti-CD28 mAbs (cross-hatched bar; experiments done as in the middle trace of **A**). The asterisks indicate *p* < 0.0001 for Dunnett's tests compared to the proliferation of the CD3/CD28 stimulated T cells (cross-hatched bar). At E:T ratios of 1:1 or more BM-MDSCs significantly suppressed T cell proliferation. **(C)** Suppression assays were done using BM-MDSCs plus CD3/CD28 stimulated T cells (5E:1T ratios as in **B**), and T cell proliferation in the absence of CRP or in the presence of 1–50 μg/ml human CRP was measured. Addition of human CRP dose-dependently enhanced the ability of mouse BM-MDSCs to suppress T cell proliferation. The *IC*_50_ and associated 95% confidence interval estimated by non-linear regression analysis is indicated (*r*^2^ = 0.551, DF = 24 obtained from two separate co-cultures each done in triplicate). **(D)** Representative CFSE dilution histograms (normalized to mode) for mouse CD4^+^ T cells stimulated with anti-CD3 and anti-CD28 mAbs; from bottom to top the T cells received no further treatment, BM-MDSCs (1E:20T), enriched DCs (immunomagnetically positively-selected; 1E:20T ratio; see *Materials and Methods* and Supplemental Figure 1), or enriched MDSCs (immunomagnetically negatively-selected; 1E:20T ratio. See *Materials and Methods* and Supplemental Figure 1). Note that the proliferation of T cells is effectively suppressed by the enriched MDSCs (top trace), whereas it is effectively enhanced by the enriched DC fraction. **(E)** CFSE dilution histograms (normalized to mode) for mouse CD4^+^ T cells stimulated with anti-CD3 and anti-CD28 mAbs. From bottom to top the T cells received: no further treatment, enriched DCs, enriched DCs plus human CRP, enriched MDSCs, or enriched MDSCs plus CRP. When MDSCs and CRP were added they were added at 1E:20T and 100 μg/ml, respectively. Note that addition of CRP only enhanced the suppressive capacity of enriched MDSCs (top trace). **(F)** CD4^+^ T cell proliferation indices (normalized to CD3/CD28 stimulated T cells; cross-hatched bar. See the *Materials and Methods*) for the representative experiment shown in **(E)**. The asterisk indicates significantly more suppression of T cell proliferation (*p* < 0.05 for Sidak's test) in the presence of enriched MDSCs plus CRP than in the presence of enriched MDSCs without CRP.

To establish that the BM-MDSCs we generated are *bona fide* suppressor cells and to test if CRP influences their suppressive activity, we used mouse BM-MDSCs as effector cells (E) in co-culture assays with CD3/CD28 stimulated target (T) mouse CD4^+^ T cells. At E:T ratios of 1:1, 5:1, and 10:1, unfractionated BM-MDSCs significantly suppressed the proliferation of T cells (Figures 3A,B). Importantly, at a 5:1 E:T ratio human CRP dose-dependently augmented mouse BM-MDSC mediated suppression of mouse CD4^+^ T cell proliferation with an *IC*_50_ of 1.165 μg/ml (Figure 3C). In the absence of BM-MDSCs human CRP (≤ 100 μg/ml) had no discernable effect on the proliferation of mouse CD4^+^ T cells (Supplemental Figure 2C).

To verify that the observed suppression of CD4^+^ T cell proliferation was attributable to the action of MDSCs *per se* and not to other potentially suppressive cells present in the mouse BM-MDSC cultures, we compared the suppressive capacity of DCs (CD11c^+^ cells captured by immunomagnetic selection) vs. enriched MDSCs (CD11c^−^ cells left behind after immunomagnetic selection that are ~94% pure MDSCs as shown in Supplemental Figures 1E,F) from the same BM cultures. We found that after their enrichment in this way, mouse MDSCs were capable of fully suppressing mouse CD4^+^ T cell proliferation even at an E:T ratio of 1:20 (Figure 3D). In stark contrast, at a 1:20 E:T ratio the DCs promoted T cell proliferation rather than suppressed it (Figure 3D). Importantly, human CRP (100 μg/ml) significantly enhanced the suppressive actions of enriched mouse MDSCs but had no significant effect on the actions of mouse DCs (Figures 3E,F). These data confirm that the mouse myeloid-derived cells we generated are *bona fide* MDSCs and that human CRP selectively enhances their suppressive capacity.

### In the Absence of FcγRIIB Human CRP Does Not Augment Mouse MDSC Mediated Immune Suppression

**Figure 4:**
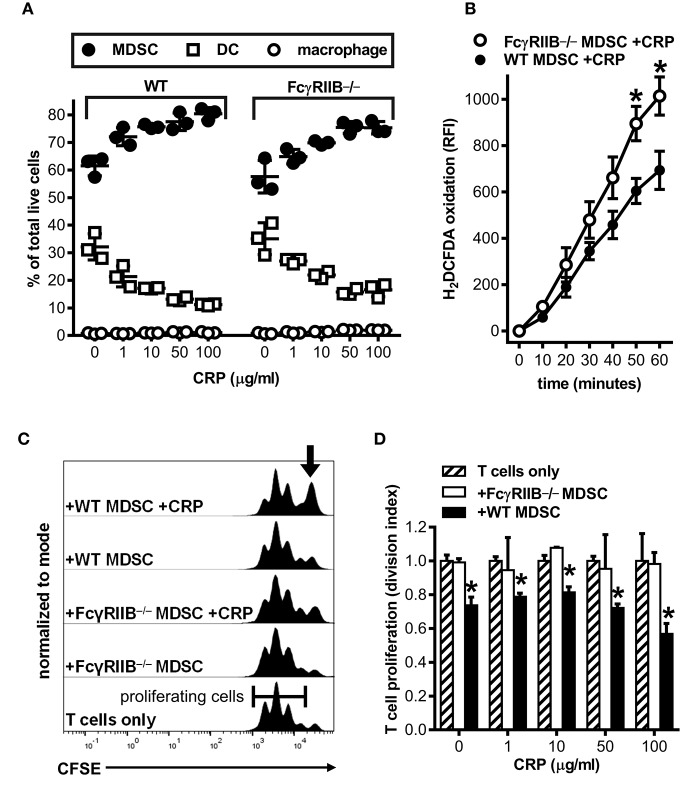
Human CRP does not enhance the suppressive capacity of mouse FcγRIIB^−/−^ MDSCs. Mouse MDSCs were generated from bone marrows supplied by wild type (WT) versus FcγRIIB^−/−^ mice to study the receptor's requirement for the generation of MDSCs and to assess its requirement for CRP dependent production of iROS and suppression of T cell proliferation. **(A)** The absence of FcγRIIB expression has no impact on human CRP's ability to drive MDSC generation. The data shown (mean ± SD) are representative of *n* = 4 separate cultures each conducted in triplicate (compare to Figure 1G). **(B)** The absence of FcγRIIB expression increases CRP triggered (100 μg/ml) iROS generation by enriched MDSCs. The data shown (mean ± SEM) are pooled across three separate cultures each conducted in triplicate. The asterisks indicate *p* < 0.05 for unpaired *t*-tests compared to time-matched WT. **(C)** Representative CFSE dilution histograms (normalized to mode) for WT mouse CD4^+^ T cells stimulated with anti-CD3 and anti-CD28 mAbs; from bottom to top the T cells received no other treatment, enriched FcγRIIB^−/−^ MDSCs, enriched FcγRIIB^−/−^ MDSCs plus human CRP, enriched WT MDSCs, or enriched WT MDSCs plus human CRP. When MDSCs and CRP were added they were added at 1E:5T and 100 μg/ml, respectively. Note that addition of CRP only enhanced the suppressive capacity of enriched MDSCs (indicated by the increased proportion of undivided T cells shown by the arrow in the top trace). **(D)** T cell proliferation (division indices; see the *Materials and Methods*) for the representative mean + SD of two co-cultures conducted in triplicate of **(C)**. The asterisks indicate significantly lower division indices (*p* < 0.05 for Dunnett's tests) at each dose of CRP when using WT MDSCs vs. no MDSCs.

Many of the reported effects of human CRP on myeloid cells *in vitro* and *in vivo* have been attributed to CRP utilization of various FcRs, and there is much evidence for CRP utilizing the inhibitory Fc gamma receptor IIB (FcγRIIB, CD32B) (7, 8, 30). Since FcγRIIB can operate in *trans* to inhibit the actions of activating receptors like FcγRI (CD64) and FcγRIII (CD16) (31, 32), we used bone marrow from FcγRIIB^−/−^ mice (24) to determine whether CRP-mediated augmentation of mouse MDSC expansion, iROS production, and CD4^+^ T cell suppressive function might require FcγRs. First we verified that wild type mouse MDSCs express FcγRI, IIB, and III (flow cytometry data not shown), confirmed that cultures of FcγRIIB^−/−^ BM yielded similar cell numbers with comparable viability compared to wild type BM cultures (Table 1) and that the relative proportions of MDSCs, DCs, and macrophages generated in FcγRIIB^−/−^ BM cultures was similar to that in wild type BM cultures (Figure 4A), and showed that absence of FcγRIIB did not alter the ability of mouse MDSCs to mount a respiratory burst or produce iROS after PMA stimulation (Supplemental Figure 3). Next we tested the influence of CRP on wild type vs. FcγRIIB^−/−^ cells. We found that human CRP enhancement of mouse MDSC generation at the expense of DCs was largely similar for FcγRIIB^−/−^ compared to wild type (Figure 4A). However, the production of iROS by enriched MDSCs stimulated with human CRP (100 μg/ml) was significantly greater for FcγRIIB^−/−^ than for wild type (Figure 4B). Despite this, human CRP did not enhance the ability of FcγRIIB^−/−^ MDSCs to suppress mouse CD4^+^ T cell proliferation (Figures 4C,D). These data suggest that CRP's ability to potentiate the immune suppressive actions of MDSCs is likely modulated both directly and indirectly by FcγRs.

**Table 1:** Yields from cultures of wild type vs. FcγRIIB^−/−^ mouse bone marrow.

	**Wild type**	**FcγRIIB^**-/-**^**	**One-tailed**
	**Mean ± SEM *(n*^**a**^*)***	**Mean ± SEM *(n*^**a**^*)***	**Paired *t*-test**
% Viable cells in culture^b^	97.5 ± 0.6 (4)	97.7 ± 0.3 (4)	*p* = 0.3064
Number viable cells in culture^c^	1.88 ×10^6^ ± 7.6 ×10^5^ (3)	2.21 ×10^6^ ± 1.1 ×10^6^ (3)	*p* = 0.2207
Number MDSCs^d^	1.34 ×10^6^ ± 4.0 ×10^5^ (3)	1.05 ×10^5^ ± 5.3 ×10^5^ (3)	*p* = 0.1160

a *Number of separate cultures interrogated*.

b *Percent of viability dye eFlour780^low^ cells in d4 cultures, estimated by flow cytometry. See Materials and Methods*.

c *Number of trypan-blue negative cells in d4 cultures, counted on a hemocytometer. See Materials and Methods*.

d *Number of trypan blue negative MDSCs in d4 cultures after magnetic removal of CD11c^+^ cells, counted on a hemocytometer. See Materials and Methods*.

### Human CRP Enables Primary Human Neutrophils to Suppress Proliferation of Autologous T Cells

**Figure 5:**
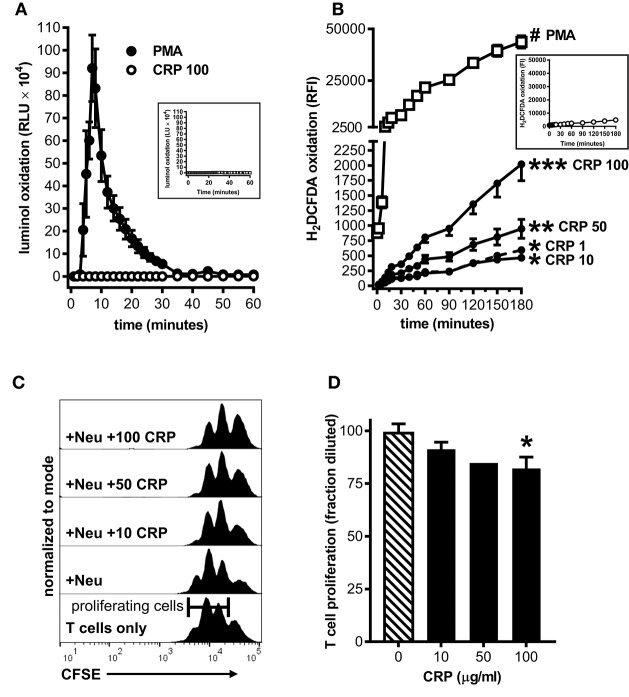
Human CRP triggers iROS production by human peripheral blood neutrophils and grants them the capacity to suppress the proliferation of autologous T cells. Peripheral blood neutrophils were isolated from five healthy human donors (see the *Materials and Methods*) to assess CRP's impact on their production of iROS and suppression of T cell proliferation. **(A)** ROS generated by human neutrophils stimulated with PMA (100 nM) vs. human CRP (100 μg/ml) was measured by luminol assay. Similar to mouse MDSCs (see Figure 2A), PMA stimulated human neutrophils showed a strong respiratory burst and those stimulated with CRP did not. Untreated controls are shown in the inset. **(B)** Intracellular ROS generated by human neutrophils stimulated with PMA (100 nM) vs. human CRP (1–100 μg/ml) was measured with the cell-permeant redox-sensing fluorescent dye H_2_DCFDA. Similar to mouse MDSCs (see Figure 2B), human neutrophils exhibited a significant increase in iROS-dependent fluorescence intensity when stimulated with 100 nM PMA or high concentrations of human CRP. The data shown are the mean ± SEM for two donors each done in triplicate and the asterisks indicate significantly higher RFI than untreated controls (shown in the inset) by Sidak's multiple comparisons tests; ^*^p < 0.05 at 60 min, ^**^p < 0.05 at 30 min, and ^***^p < 0.05 at 14 min for CRP, and ^#^*p* < 0.05 at 12 min for PMA. **(C)** Representative CFSE dilution histograms (normalized to mode) for autologous human CD3^+^ T cells stimulated with anti-CD3 and anti-CD28 mAbs. From bottom to top the T cells received no further treatment, neutrophils (Neu at 1E:1T), or Neu plus 10–100 μg/ml human CRP. The addition of CRP bestowed suppressive capacity to the autologous neutrophils in a dose-dependent fashion, as indicated by the rightward shift in the CFSE histograms. **(D)** CD4^+^ T cell proliferation in the presence of Neu performed as in **(C)**. The data shown are mean + SEM pooled across donors and are normalized to CD3/CD28 stimulated T cells in the presence of Neu with no CRP added (cross-hatched bar). The asterisk indicates significantly more suppression of T cell proliferation (*p* < 0.05 for Sidak's test) in the presence of CRP than in its absence.

To ascertain the potential clinical relevance of our findings we sought evidence that human CRP also promotes an immune suppressive phenotype in human myeloid lineage cells. Because under certain conditions (such as cancer and severe injury) mature neutrophils can act as MDSCs (33), and because large numbers of them are easily obtained from the circulation, we isolated peripheral blood neutrophils from five healthy human donors for these studies. Like mouse MDSCs, human neutrophils treated with human CRP did not exhibit a respiratory burst (compare Figures 2A, 5A). Also like mouse MDSCs, human neutrophils treated with human CRP exhibited CRP dose-dependent production of iROS (compare Figures 2B, 5B). When co-cultured with autologous CD3/CD28 stimulated human CD3^+^ T cells (1:1 E:T ratio) the neutrophils *per se* did not significantly impact T cell proliferation, but importantly in the presence of increasing amounts of human CRP they significantly suppressed it (Figures 5C,D). These data show that—like mouse MDSCs—human CRP grants suppressive capacity onto human primary blood neutrophils.

## Discussion

The genes encoding mouse and human CRP have very similar nucleotide sequences and genomic organization (34) and the mouse and human proteins share at least 70% amino acid sequence identity (35). The biological activity of mouse CRP has been the subject of comparatively few direct investigations [e.g., (36)], but because of this high homology it is generally assumed that mouse CRP has biological actions similar to that of human CRP (ability to activate complement, ability to bind FcRs, etc.). Importantly, because mouse CRP is not a major acute phase reactant (i.e., CRP concentration in the circulation of mice remaining below ~3 μg/ml under all conditions), CRPtg mice have been widely used to study the impact of the human CRP acute phase response *in vivo*. For example in our prior studies we established that the outcome of ischemic AKI is worse for CRPtg compared to wild type mice, linked this effect to the human CRP acute phase response and its associated heightening of renal infiltration by MDSCs during AKI (14, 15). We also showed that the severity of AKI could be lessened by (i) reducing MDSC infiltration with an anti-Gr-1 antibody (15) or (ii) by targeting human CRP with an antisense oligonucleotide (37). These and other findings suggested that during AKI in mice, CRP promotes MDSC generation/expansion/renal infiltration and thereby propels the injury response; this effect is more pronounced in CRPtg because of their high levels of human CRP.

Expansion and infiltration of myeloid cells at the sites of injury and their acquisition of MDSC phenotypes and functions is well-described in the settings of cancer, trauma, and sepsis (16, 28). MDSC expansion is thought to be achieved by a shift in the hematopoietic pool toward granulocyte and monocyte progenitors (i.e., precursors of neutrophils and monocytes/macrophages/DCs, respectively), thereby increasing the pool of effector myeloid cells available for quick resolution of the insult (38). Thus, during both acute (e.g., AKI) or chronic (e.g., cancer) pathological states, this response is thought to yield an expansion of myeloid-derived cells that exhibit a strong suppressor function: the so-called MDSC. MDSCs are generally considered immature cells since they lack the nuclear morphologies and/or the portfolio of surface markers characteristic of mature neutrophils, macrophages, and DCs. Confounding this interpretation are reports that MDSCs can “mature” into neutrophils and macrophages within the same pathological milieu thought to promote their initial expansion (39, 40). These observations support the counter-hypothesis that MDSCs are not a unique cell type derived from dedicated MDSC progenitors in the BM, but rather are mature leukocytes that acquire an atypical suppressive function in the periphery (16, 38). Notwithstanding this uncertainty and even in the absence of a specific marker for their identification and purification, *bona fide* MDSCs should have a T cell suppressive action.

Despite the present uncertainty about their true origin, identity, and developmental fate, we show herein that CRP can promote the expansion of mouse BM myeloid derived cells and enhance their suppressive phenotype. Furthermore, we show that exposure to CRP bestows upon mature human neutrophils a suppressive phenotype. The ability of CRP to promote MDSC expansion from mouse BM is likely related to CRP's ability to selectively increase the cell-cycling of MDSC progenitors, as CRP had no effect on the overall rate of cell death in BM cultures. Furthermore, even low doses of human CRP potentiated mouse BM progenitor commitment toward MDSCs and steered them away from DCs. These observations are in alignment with our recent report that human CRP also inhibited the generation of mouse DCs in a completely different *in vitro* system (29). The ability of human CRP to promote mouse MDSC expansion on one hand, while inhibiting mouse DC expansion on the other, suggests that CRP might be a tonic regulator of BM progenitor lineage commitment and expansion—particularly during inflammation when the amount of human CRP is elevated. Likewise others have shown that human CRP can increase expression of CD206 (a marker of anti-inflammatory polarization) on monocytes, but not fully differentiated macrophages, also suggesting that CRP has more of an impact on less differentiated myeloid cells (41). In their sum, our latest findings suggest that (at least in mice) human CRP promotes the differentiation of myeloid progenitors into effector cells with suppressor functions, meanwhile dampening the development of myeloid cells that would otherwise promote adaptive immunity. Importantly, human CRP also evokes suppressive actions from human neutrophils.

Perhaps foreshadowing our findings by nearly 4 decades, Marcelletti et al. (42) reported that CRP potentiated monocytopoiesis by acting on FcR-expressing mouse myeloid progenitor cells in S-phase. FcRs are categorized based on their inhibitory or activating signaling potential and many of them are known to be utilized by CRP. Notably, the potent inhibitory FcR, FcγRIIB (CD32B), is used by CRP in both mice and humans (8, 29, 43–45). In this report we show that CRP does not rely on FcγRIIB to alter BM progenitor lineage commitment toward MDSCs. This outcome is similar to that reported by others, who showed that CRP can promote the generation of inflammatory macrophages from mouse BM even in the absence of FcγRs (46). Nevertheless, our results show that FcγRIIB is involved in CRP triggered ROS production by MDSCs, and CRP requires FcγRIIB to promote the suppressive function of MDSCs. Additional studies are needed to fully explore the contribution of other FcRs that CRP might utilize, e.g., activating FcγRs (8) and the activating FcαR (30). The latter is of particular interest as recently it was shown that engagement of FcαR, as opposed to engagement of FcγR, more potently stimulates human neutrophils to kill cancer cells (47); CRP may be one of the FcR ligands mediating this effect. Additionally, further in depth research will be needed to thoroughly investigate the influence of CRP on myeloid lineage development.

One of the most potent suppressive mechanisms in the armamentarium of MDSCs is their ability to produce high amounts of ROS, whether derived from superoxide generated by membrane-bound NADPH-oxidases, the endoplasmic reticulum, or the mitochondrial electron transport chain. In our hands, CRP did not trigger a respiratory burst from mouse BM-MDSCs or human neutrophils, but did stimulate a monophasic increase in ROS consistent with their production of iROS. Furthermore, the production of iROS achieved statistical significance only when high concentrations of CRP were used, i.e., levels of CRP consistent with those found during inflammation (2). Although CRP stimulated a greater increase in iROS for FcγRIIB^−/−^ than wild type mouse MDSCs (Figure 4B), CRP triggered iROS production by mouse MDSCs lacking all activating FcγRs (48) was not different than wild type MDSCs (data not shown). Taken together these findings are consistent with the notion that, during inflammatory episodes when CRP is elevated, CRP stimulates iROS production by mouse MDSCs and this is tempered by FcγRIIB engagement.

Despite being dispensable for CRP mediated enhancement of *in vitro* generation of MDSCs, FcγRIIB appears essential for CRP mediated promotion of their suppressive actions on T cells. To explain this seemingly paradoxical situation we are currently investigating the possibility that conversion of superoxide to hydrogen peroxide, a cell permeant ROS, is impaired in FcγRIIB^−/−^ MDSCs, perhaps due to decreased expression of antioxidants such as superoxide dismutase. Accordingly, in WT MDSCs the interaction of CRP with FcγRIIB might increase the expression of antioxidants, allowing for increased conversion of superoxide to hydrogen peroxide and thereby promoting the immune suppressive action of MDSCs. In the absence of FcγRIIB this pathway would be eliminated, allowing for accumulation of superoxide and other ROS but reduced conversion to hydrogen peroxide. In support of this model others have shown that superoxide-derived hydrogen peroxide generated by MDSCs is responsible for suppression of T cell activation and proliferation (49). Alternatively, CRP might regulate antioxidant gene expression by modulating the expression/action of the transcription factor Nuclear factor (erythroid-derived 2)-like 2 (Nrf2), which is known to be highly expressed by MDSCs and thought to allow them to withstand the high oxidant stresses experienced during their expansion (50, 51). We are currently investigating this possibility. Also since M-MDSCs also produce RNS (52, 53), we are investigating whether CRP impacts MDSC generation of RNS.

The majority of renal MDSCs recovered from CRPtg mice subjected to AKI (15) and the majority of MDSCs generated *in vitro* from mouse BM precursors (this study) are of the PMN-MDSC subtype. Since the effects of human CRP on mouse MDSCs might be an aberration of a human protein/mouse cell system, and given the ongoing debate about PMN-MDSCs as a distinct lineage vs. neutrophils that gain suppressive functions in the periphery (33, 54), we sought to determine whether human CRP had the same effects on human neutrophils as it did on mouse MDSCs. Like mouse MDSCs, CRP-treated human neutrophils did not exhibit a respiratory burst but did show a dose-dependent increase in their production of iROS. Most importantly, exposing human primary neutrophils to human CRP rendered them capable of suppressing the proliferation of autologous CD3^+^ T cells. An important caveat is that in our suppression assays we used mouse CD4^+^ T cells vs. human CD3^+^ T cells (the latter comprised of 64.7 ± 3.8% CD4^+^ cells and 28.7 ± 4.2% CD8^+^ cells, *n* = 2 donors), so the two *in vitro* systems and the magnitude of the CRP effects therein cannot be compared directly. Nevertheless, the similarity in the effect of human CRP on mouse MDSCs vs. human neutrophils suggests that monitoring and targeting CRP might be a valid clinical strategy for overcoming MDSC/neutrophil mediated immune suppression. For example, CRP blood levels could be lowered using various available methods such as an antisense-oligonucleotide to CRP (37), small-molecule inhibitors of CRP (55), or apheresis of CRP (56). Either of these CRP-lowering approaches might re-establish homeostatic hematopoiesis and/or foster the development of beneficial myeloid lineages. Consequently, patients with aberrant or over-represented pathologic myeloid effectors, such as those with cancer, AKI, etc., might benefit from specific lowering of CRP.

## Data Availability

The datasets generated for this study are available on request to the corresponding author.

## Ethics Statement

This study was carried out in accordance with the recommendations of the *Guide for the Care and Use of Laboratory Animals Eighth Edition* (NIH Academies Press, 2011), the Institutional Animal Care and Use Committees, and Institutional Review Board at the University of Alabama at Birmingham.

## Author Contributions

RJ and AS conceptualized this study, wrote the manuscript, conducted formal analysis, and data visualization. RJ, AS, and ZH reviewed and edited the manuscript. RJ, VK, AC, ZH, and AS designed experiments. RJ, VK, and AC conducted experiments.

### Conflict of Interest Statement

The authors declare that the research was conducted in the absence of any commercial or financial relationships that could be construed as a potential conflict of interest.

## Abbreviations

CRP: C-reactive protein
CRPtg: human C-reactive protein transgenic mice
MDSC: myeloid derived cells with a suppressor phenotype
BM: bone marrow
BM-MDSC: bone marrow generated myeloid derived cell with suppressor functions
PMN-MDSC: polymorphonuclear-MDSC
M-MDSC: monocytic-MDSC
DC: dendritic cell
iROS: intracellular reactive oxygen species
RNS: reactive nitrogen species
E:T: Effector to Target ratio
FcR: Fc receptor
FcγRIIB: Fc gamma receptor IIB
AKI: acute kidney injury
mAb: monoclonal antibody
H_2_DCFDA: 2′,7′-dichlorodihydrofluorescein diacetate
7-AAD: 7-aminoactinomycin D
RLU: relative luminescence unit
RFI: relative fluorescence intensity
Nrf2: nuclear factor erythroid 2-related factor 2.
