# Supplementary material for: C-Reactive Protein Promotes the Expansion of Myeloid Derived Cells With Suppressor Functions
Source: Front Immunol. 2019 Sep 18;10:2183. doi: 10.3389/fimmu.2019.02183 (PMC6759522; doi:10.3389/fimmu.2019.02183)
Supplement: Supplementary file 1 [file Data_Sheet_1.docx]

Supplementary Material

**A**

**C**

**F**

**H**

**D**


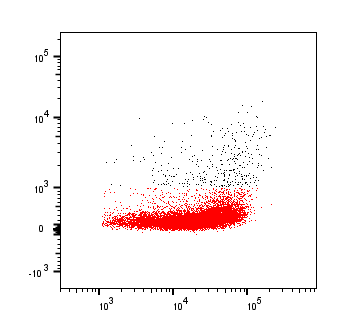


**B**

**E**

**G**

d4 BM cultures


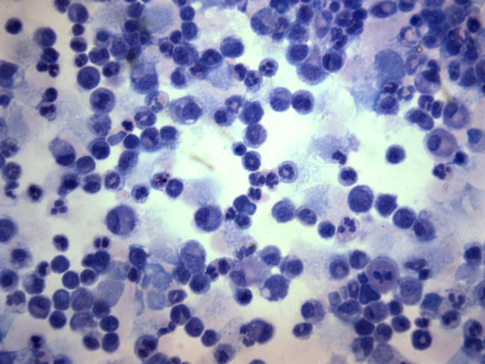


magnetic selection

**CD11b**


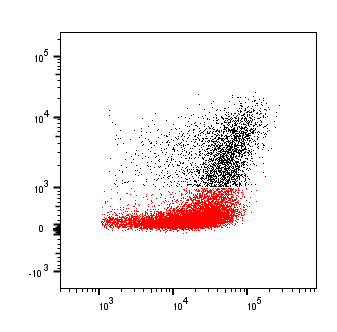


**CD11c**


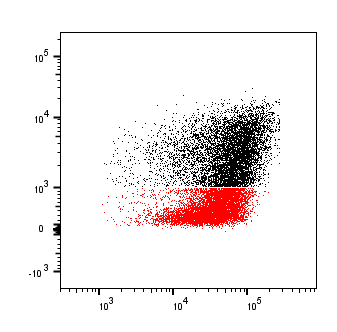


**CD11c**

**MDSC**

45.7%

**DC**

51.6%

**CD11c**

**MDSC**

94.9%

**DC**

2.2%

**MDSC**

78.1%

**DC**

18.6%

**Supplemental Figure 1. Enrichment of MDSCs from mouse bone marrow cultures.** Mouse bone marrow (BM) progenitors were cultured under conditions tailored to generate MDSCs (1). On day 4 of culture the cells (**A**) were subjected to flow cytometry to enumerate CD11c^‒^CD11b^+^F4/80^‒^Ly6G^+^Ly6C^+^ MDSCs and CD11c^+^CD11b^+^ DCs (**B**). MDSCs represented 79.2 ± 1.8% of all live cells in the d4 BM cultures (**C**). Samples from the same cultures were subjected to immunomagnetic selection (see the *Materials and Methods*) and the number of CD11c^+^ cells (DCs; positively selected) and CD11c^‒^ cells (MDSCs; negatively selected) were enumerated using a hemocytometer. CD11c^‒^ cells represented 76.7 ± 7.1% of all live cells in the d4 BM cultures (n = 7 cultures; panel **D**). Flow cytometry of the negatively selected cells (**E**, **F**) confirmed that the CD11c^‒^ CD11b^+^F4/80^‒^Ly6G^+^Ly6C^+^ MDSCs were highly enriched (94.0 ± 1.5% of all live negatively selected cells). Note the higher frequency of MDSCs following enrichment (F) compared to the BM-MDSC culture prior to immunomagnetic selection (C). Panels **G** and **H** show that the positively selected fraction is a mixture of DC and MDSCs.

**B**


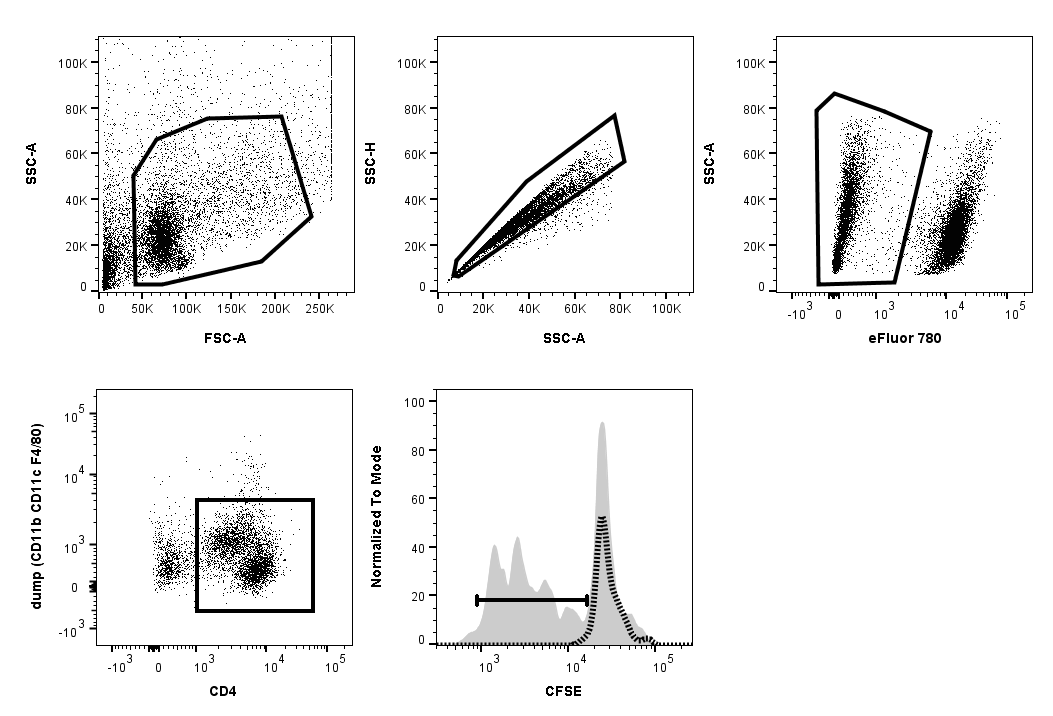


stimulated

unstimulated

**A**

**C**


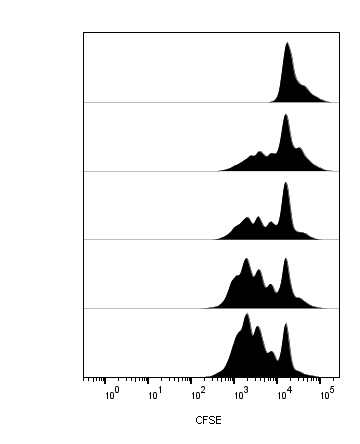


**0 μg/ml**

**0.25 μg/ml**

**1 μg/ml**

**2 μg/ml**

**4 μg/ml**

**normalized to mode**

**CFSE**

**D**


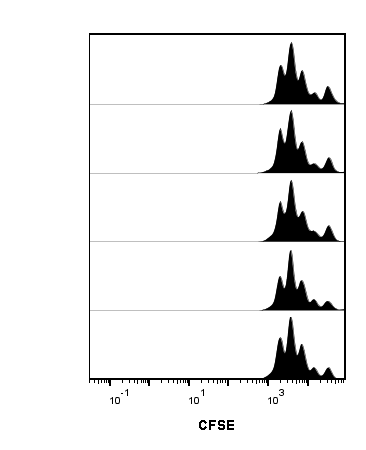


**100 CRP**

**50 CRP**

**10 CRP**

**1 CRP**

**0 CRP**

**normalized to mode**

**CFSE**

Supplemental Figure 2. Strategy for identification of proliferating CFSE^+^CD4^+^ T cells in BM-MDSC:T cell co-cultures. Three days after initiating co-cultures of BM-MDSCs plus CD3/CD8 stimulated CFSE-labeled T cells the cells were harvested and processed for flow cytometry. (A) Sequentially (top row of panels from left to right), debris was gated out using a FSC-A × SSC-A plot, doublets were gated out using a SSC-A × SSC-H plot, and eFluor780^+^ dead cells were gated out. Next using a CD11b^+^CD11c^+^ F4/80^+^ dump gate myeloid cells were excluded (B). The gated CD4^+^ T cells were then used to generate CFSE histograms (normalized to mode) and unstimulated versus CD3/CD28 stimulated T cells were compared to identify and enumerate proliferating T cells (indicated by the horizontal bracket). Prior to performing T cell proliferation assays each lot of anti-CD3 mAb was titrated and used at concentrations that resulted in 3 to 5 discernable generations of CFSE^+^CD4^+^ T cells after 72 h of culture; panel C shows representative CFSE histograms for T cells stimulated with increasing amounts (0 – 4 μg/ml) of anti-CD3 mAb. (D) In preliminary experiments we verified that (in the absence of BM-MDSCs) human CRP (1-100 μg/ml) had no discernable effect on the proliferation of CD3/CD28 stimulated mouse CD4^+^ T cells.

**C**

**B**

**A**

Supplemental Figure 3. Loss of FcγRIIB does not significantly alter PMA-induced production of ROS by mouse MDSCs. Mouse MDSCs were generated from bone marrow (BM) supplied by wild type (WT) or FcγRIIB^-/-^ mice to study the receptor’s requirement for the generation of ROS. The generation of ROS after stimulation with PMA (100 nM) was not affected by loss of FcγRIIB expression. Extracellular ROS production by (A) BM-MDSCs or (B) enriched MDSCs measured via luminol assay and (C) intracellular ROS production by enriched MDSCs measured via H_2_DCFDA assay.

# References

1. Höchst B, Mikulec J, Baccega T, Metzger C, Welz M, Peusquens J, et al. Differential induction of Ly6G and Ly6C positive myeloid derived suppressor cells in chronic kidney and liver inflammation and fibrosis. *PloS one* (2015) 10(3):e0119662. doi: 10.1371/journal.pone.0119662. PubMed PMID: 25738302; PubMed Central PMCID: PMC4349817.
